# Acute compartment syndrome of the lower leg causing cardiac arrest after resection of the right external iliac vein for autologous graft: a case report

**DOI:** 10.1186/s40981-019-0286-2

**Published:** 2019-10-23

**Authors:** Koji Hoshino, Toru Nakamura, Mineji Hayakawa, Yusuke Itosu, Hitoshi Saito, Satoshi Hirano, Yuji Morimoto

**Affiliations:** 1 Department of Anesthesiology, Hokkaido University Hospital, N14W5, Kita-ku, Sapporo, 0608648 Japan; 2 Department of Gastroenterological Surgery II, Hokkaido University Faculty School of Medicine, N15W7, Kita-ku, Sapporo, Hokkaido 0608638 Japan; 3 Emergency and Critical Care Center, Hokkaido University Hospital, N14W5, Kita-ku, Sapporo, 0608648 Japan

**Keywords:** Acute extremity compartment syndrome, Portal vein reconstruction, Hyperkalemia

## Abstract

**Background:**

The right external iliac vein (REIV) is often used for portal vein reconstruction in patients undergoing pancreatoduodenectomy with portal-superior mesenteric vein resection. We report a case of cardiac arrest caused by acute lower leg compartment syndrome as a result of REIV resection.

**Case presentation:**

A 53-year-old man underwent pancreatoduodenectomy with portal vein resection. Hyperkalemia progressed during surgery due to intestinal reperfusion injury, which caused recurrent ventricular arrhythmia required for cardio-pulmonary resuscitation. The surgery was discontinued after resuscitation, and portal vein reconstruction using the REIV was performed 2 days post-operatively. Acute compartment syndrome was diagnosed immediately following the surgery. Hyperkalemia progressed, causing pulseless ventricular tachycardia. Emergent fasciotomy was performed, but right leg dysfunction persisted after discharge.

**Conclusion:**

REIV resection can cause lower-extremity acute compartment syndrome. The status, including intracompartmental pressure, of the lower extremity should be carefully observed after REIV resection during and after surgery.

## Background

Portal-superior mesenteric vein resection and reconstruction with autologous vein graft is generally performed for patients with pancreatobiliary malignancies [1]. Different vein types are used as autologous grafts, such as the left renal vein [2], internal jugular vein [3], and external iliac vein [4]. The right external iliac vein (REIV) is satisfactory in both diameter and length for grafting; Schanzer et al. first reported that donor extremities had minimal disability and swelling after surgery when using REIV grafts [4]. Although Kaneoka et al. recently reported that right leg symptoms after REIV resection for autologous grafts could be unexpectedly prolonged [5], no reports about life-threatening complications after REIV resection have been published. Herein, we report a case of acute lower leg compartment syndrome after REIV resection, causing cardiac arrest due to hyperkalemia.

## Case presentation

A 53-year old man (height, 175 cm; weight, 87 kg) was scheduled to undergo subtotal stomach-preserving pancreatoduodenectomy with portal vein reconstruction for pancreatic cancer after receiving neoadjuvant chemotherapy with gemcitabine and nab-paclitaxel. Laparoscopic left nephrectomy for renal cell carcinoma was performed 1 year prior; therefore, harvesting the left internal jugular vein for portal vein reconstruction was planned because the left renal vein was too short. Preoperative serum creatinine level and potassium level were 1.39 mg/dL and 4.1 mEq/L, respectively.

General anesthesia was induced rapidly with propofol, rocuronium, fentanyl, and remifentanil and maintained with desflurane. The patient’s vital signs remained stable while resection of the distal stomach, gallbladder, distal common bile duct, and pancreatic head was performed. Bleeding from the first jejunal vein (J1V) occurred during adhesion detachment around the superior mesenteric vein (SMV), and clamping of the J1V was required to control the bleeding. Immediately after J1V clamping, bowel congestion progressed because the patient’s intestinal blood flow had been bypassed from the SMV to the inferior pancreaticoduodenal vein through J1V due to complete obstruction of the portal vein by the tumor. Therefore, a heparin-coated bypass tube (ANTHRON™ bypass tube, TORAY MEDICAL Co., Tokyo, Japan) was inserted from the SMV to the right great saphenous vein to relieve the bowel congestion. Several minutes after insertion of the ANTHRON™ bypass tube, blood potassium level rose from 4.7 mEq/L to 6.0 mEq/L, and pulseless ventricular tachycardia occurred. Chest compression was promptly performed by surgeons, and anesthesiologists attempted direct current defibrillation several times; epinephrine, magnesium sulphate, bicarbonate, lidocaine, and amiodarone were also administered. In addition to resuscitation, anesthesiologists inserted a blood-access catheter to the right internal jugular vein and started continuous renal replacement therapy to prevent elevation of blood potassium level. Spontaneous circulation was recovered after 30 min. The surgical team decided to discontinue the surgery, and the patient was transferred to the intensive care unit (ICU) after packing gauze into the abdomen and with the ANTHRON™ bypass tube left inserted. Total blood loss was 5620 mL, and total fluid balance was + 6115 mL. The operation time was 10 h 30 min.

Two days after the first operation, the patient was scheduled to undergo tumor resection and portal vein reconstruction with REIV. The surgical team abandoned using the left internal jugular vein for portal vein reconstruction because of concerns that brain congestion after harvesting the jugular vein could affect brain function recovery. Anesthesia was maintained with dexmedetomidine, fentanyl, rocuronium, and remifentanil. Continuous hemodiafiltration had been performed in the ICU due to persistent anuria and continued during the operation to prevent hyperkalemia. Both legs were compressed with a pneumatic compression device throughout the operation to prevent deep venous thrombosis formation. A section of the REIV 5 cm in length was harvested intraperitoneally with a surgical stapler, and the REIV distal end was closed with a 4-0 polypropylene running suture. The inferior epigastric vein, pubic branch of the obturator vein, and the deep circumflex iliac vein were preserved. A surgical nurse noticed swelling of the patient’s right leg 2 h after harvesting; the surgical team ensured careful observation throughout the surgery because a transient change by congestion of the right leg was suspected. Blood potassium level was slightly elevated after REIV resection, but ventricular arrhythmia was absent. Surgery was completed as scheduled (operation time, 7 h 3 min), and the patient was transferred to the ICU again. Total blood loss and total fluid balance were 2485 mL and + 2587 mL, respectively.

**Fig. 1:**
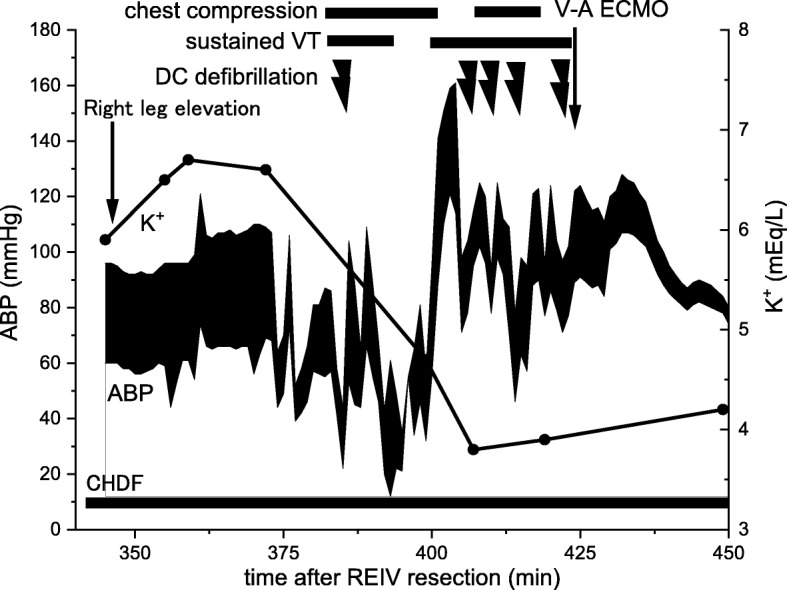
Time course in the intensive care unit after right external iliac vein resection. Blood potassium level was rapidly elevated after elevating the right leg. ABP arterial blood pressure, CHDF continuous hemodiafiltration, DC direct current, V-A ECMO veno-arterial extracorporeal membrane oxygenation, REIV right external iliac vein

On ICU admission, distention and fullness of the right leg worsened. An orthopedist diagnosed the patient with acute compartment syndrome, and right leg elevation to lower the intracompartmental pressure was initiated. Blood potassium level was rapidly elevated immediately after leg elevation (Fig. 1), which caused sustained ventricular tachycardia despite efforts to lower blood potassium level such as the administration of bicarbonate, glucose-insulin, and continuous hemodialysis with potassium-free dialysate. Chest compressions were initiated immediately. Ventricular tachycardia was prolonged; therefore, veno-arterial extracorporeal membrane oxygenation (V-A ECMO) was induced, and spontaneous circulation was recovered after resolution of hyperkalemia (Fig. 1, Table 1). Emergent fasciotomy was performed by orthopedists 1 h after induction of V-A ECMO because the intracompartmental tissue pressure of the anterior compartment was 70 mmHg.

**Table 1:** Perioperative change in arterial blood gases and compounds

	Before REIV resection		After REIV resection				After ROSC
	Preop	1 h	1 h	2 h	4 h^a^	6 h^b^	1 h
FiO_2_	0.6	0.45	0.45	0.45	0.45	0.6	0.6
pH	7.329	7.455	7.422	7.413	7.395	7.47	7.403
PaCO_2_ (mmHg)	45	36.9	39.6	38.9	41.2	33.2	37.9
PaO_2_ (mmHg)	148	170	201	197	188	137	86.5
HCO_3_^−^ (mEq/L)	23	25.6	25.4	24.4	24.7	23.9	23.2
Base excess (mEq/L)	− 2.5	2.1	1.4	0.4	0.3	1.1	− 0.8
Na^+^ (mEq/L)	138	139	137	137	135	139	141
K^+^ (mEq/L)	3.9	4.7	5.5	5.4	5.3	6.7	5.3
Ca^2+^ (mEq/L)	1.11	0.98	1.03	0.95	1.03	1.04	1.09

*Preop* at preoperative time, *PaCO*_*2*_ arterial carbon dioxide pressure, *PaO*_*2*_ arterial oxygen pressure, *REIV* right external iliac vein, *ROSC* recovery of spontaneous circulation

^a^At the end of surgery

^b^30 min before cardiac arrest

V-A ECMO was withdrawn 2 days later, and the patient was extubated 10 days after portal vein reconstruction. He had no apparent neural deficits; however, paralysis and paresthesia of the right leg persisted after discharge from the ICU 17 days post-operatively.

## Discussion

To our knowledge, this is the first report of a fatal complication after REIV resection for autologous grafts. Terasaki et al. reported that an interposition graft using the REIV for portal vein reconstruction following pancreatoduodenectomy was safe and effective [6]. However, in another study, moderate to severe outflow obstruction in the lower limbs was observed on air plethysmography in all patients after harvesting of the REIV, and such outflow obstruction in the lower limbs persisted for a long time causing symptoms such as pain upon long-duration standing [5]. After resection of the REIV, venous blood from the lower limb returns to the systemic circulation mainly through the inferior epigastric vein and great saphenous vein. Therefore, harvesting of the REIV does not usually cause the acute phase of complications, while harvesting of the deep thigh veins such as the superficial femoral vein and superficial femoropopliteal vein, which are distal to the junction of the inferior epigastric vein and the REIV, sometimes cause acute extremity compartment syndrome [7]. Initially, we were scheduled to harvest the left internal jugular vein. However, we were concerned that brain congestion might occur after internal jugular vein resection, because cardiopulmonary resuscitation was needed during the first surgery, and we could not confirm the patient’s level of consciousness until the second operation.

Acute compartment syndrome is characterized by an increase in intracompartmental pressure, leading to a decrease in perfusion pressure and hypoxemia of the tissue. Timely diagnosis is essential because irreversible changes are likely to occur when ischemia persists for more than 6 h [8]. Furthermore, rhabdomyolysis, which occurs subsequent to compartment syndrome, can cause hyperkalemia due to reperfusion injury, leading to cardiac arrest [9]. Initially, the surgeons and anesthesiologists did not suspect acute compartment syndrome after REIV resection because no reports of such complications existed. The surgical team considered the swelling intra-operatively as a transient symptom from vascular congestion. The accurate diagnosis of acute compartment syndrome under general anesthesia is challenging because its diagnosis is based primarily on clinical symptoms, such as severe pain [10]. If compartment syndrome had already occurred when the surgical nurse observed the swelling of right leg in our patient, it persisted for another 8 h before emergent fasciotomy was performed. Therefore, the right leg dysfunction that persisted even after ICU discharge is not surprising. Particular attention to the status of the leg throughout the surgery is vital for the early diagnosis of compartment syndrome.

We suspect that acute compartment syndrome occurred in our patient for the following reasons. First, there was massive bleeding during the first surgery, and substantial infusion and transfusion were required in both the operating room and ICU. In fact, the patient’s body weight before the second operation was 8 kg more than that before the first operation. Although physiological compartment pressures in adults are around 8 mmHg [10], they may have already risen due to edema before REIV resection, leading to the remarkable elevation of intracompartmental pressure following REIV resection. In their univariate analysis, Modrall et al. reported that greater intraoperative fluid resuscitation was a risk factor for fasciotomy after harvesting deep thigh veins distal to the REIV [7]. Thus, harvesting the REIV in patients who were edematous pre-operatively may be contraindicated. Moreover, they reported that lower preoperative ankle-brachial index (ABI) was also a significant risk factor for fasciotomy after deep thigh vein harvest. Therefore, the resection of the REIV should also be avoided for patients with lower ABI.

Second, in our patient, the main collaterals might have been occluded by thrombi before harvesting the REIV due to cardiac arrest in the first surgery, which likely lead to thrombus formation. In particular, the ANTHRON™ bypass tube was left inserted into the right great saphenous vein before harvesting the REIV. This might cause thrombus formation and dysfunction of venous drainage through the great saphenous vein and some branches of the femoral vein, which should work as collaterals after resection of the REIV. Therefore, REIV harvesting might not be recommended in case of using the ANTHRON™ bypass tube in the right great saphenous vein during the surgery. We speculate that right leg elevation caused venous return through minor collaterals, and in addition to potassium, some waste products that triggered ventricular arrhythmia entered the systemic circulation and provoked prolonged ventricular tachycardia.

To prevent these severe complications, we suggest the following. First, monitoring of intracompartmental pressure of the anterior compartment during surgery may be effective for early detection of compartment syndrome. If differential pressure (Δ*p* = diastolic blood pressure − intracompartmental pressure) persists below 30 mmHg, emergent fasciotomy should be considered [10]. Second, right leg elevation after resection of the REIV during surgery may avoid compartment syndrome and subsequent hyperkalemia by allowing continuous venous return and lowering intracompartmental pressure.

## Conclusion

REIV resection for autologous grafts can cause acute compartment syndrome of the right lower extremity. Careful observation of the right lower extremity after REIV resection during and after surgery is paramount to decrease the risk of adverse outcomes.

## Data Availability

Not applicable.

## Abbreviations

DC: Direct current
ICU: Intensive care unit
REIV: Right external iliac vein
V-A ECMO: Veno-arterial extracorporeal membrane oxygenation
